# Hydrophobic Modification of ZrO_2_-SiO_2_ Xerogel and Its Adsorption Properties to Rhodamine B

**DOI:** 10.3390/gels8100675

**Published:** 2022-10-20

**Authors:** Yan Liu, Jing Yang

**Affiliations:** School of Urban Planning and Municipal Engineering, Xi’an Polytechnic University, Xi’an 710048, China; liuyan@163.com

**Keywords:** methyl-modified, hydrophilic, hydrophobic, adsorption, rhodamine B, kinetic, adsorption isotherms

## Abstract

Zirconium nitrate pentahydrate (Zr(NO_3_)_4_·5H_2_O) and tetraethyl orthosilicate (TEOS) are used as the zirconium source and silicon source, respectively, and methyltriethoxysilane (MTES) as the hydrophobic modifier; the hydrophilic and hydrophobic ZrO_2_-SiO_2_ xerogels were prepared successfully. The xerogels were characterized using Fourier transform infrared spectra (FTIR), X-ray diffraction (XRD), scanning electron microscopy (SEM), and N_2_ adsorption–desorption measurement. The adsorption mechanism of hydrophobic ZrO_2_-SiO_2_ xerogels to RhB was described by the kinetic and adsorption isotherms. The results showed that the introduction of Si-CH_3_ groups can make the average pore size, BET surface area, and total pore volume of ZrO_2_-SiO_2_ xerogel increase. The hydrophobic ZrO_2_-SiO_2_ xerogel displays an adsorption capacity of 169.23 mg·g^−1^ for RhB dye at 25 °C and pH = 3. The adsorption process of hydrophobic ZrO_2_-SiO_2_ xerogel to RhB followed a pseudo–second-order kinetic model. Fitting results from the D–R model of adsorption indicate that the adsorption of RhB onto the hydrophobic ZrO_2_-SiO_2_ xerogels is mainly physical, accompanied by a spontaneous heat absorption process. The regeneration and recycling properties of hydrophobic xerogels were investigated, and their recoverability and reusability were demonstrated.

## 1. Introduction

**Figure 1:**
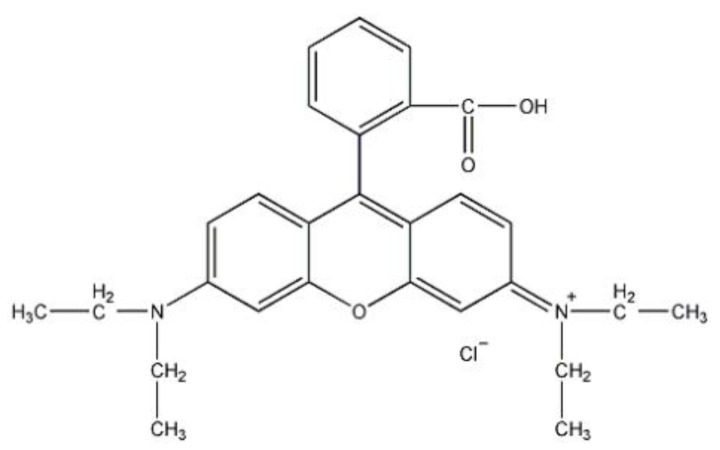
Molecular structural models of RhB.

Rhodamine B (RhB) (its structure is shown in Figure 1) is a basic cationic dye that was used in large quantities as a food additive and was later banned from the food industry because of experimental evidence of its carcinogenicity. However, RhB is still widely used in laboratories, the paper industry, the textile printing and dyeing industry, colored glass, specialty fireworks, and other industries [1]. These industries produce large amounts of RhB dye wastewater, which, if not properly treated, can cause great harm to human health and the ecological environment. Thus, it is important to seek an efficient and economical method for the treatment of dye wastewater represented by RhB [2]. Furthermore, due to the continuous industrial demand for RhB and the increased difficulty in treating RhB-containing dye wastewater, there is an increasing interest in its degradation and removal [3,4,5,6]. To our knowledge, there were many treatment methods [7,8,9,10,11] implemented for removing the eco-toxic dyes from aqueous solutions, therein including coagulation, chemical oxidation, photodegradation, membrane filtration, and adsorption. Among these methods, adsorption was deemed to be a high-efficiency and low-cost technology for removing those hazardous impurities from aqueous solutions. The high effectiveness and wide availability of an adsorbent make it very attractive for dye removal from the water environment. By far, for its low cost and energy consumption, adsorption was frequently chosen in the separation process [12,13,14]. High-performance adsorption materials mainly included activated carbon, nanometallic oxide, graphene, zeolite, “Greek coffee” grounds, etc.

Zirconia (ZrO_2_), one of the important transition metal oxides, has received considerable attention for its wide applications in oxygen sensors, fuel cell electrolytes, catalysts, and catalytic carriers, and metal-oxide-semiconductor devices due to a high number of active sites on the ZrO_2_ surface, good strength, large pore size, and high chemical stability, etc. [15]. The addition of metal oxides [16,17] can provide adsorption sites for the reactants and improve the adsorption performance, and also increase the anti-pollution properties. Ali et al. [18] prepared ZrO_2_/CeO_2_ adsorbent materials for the adsorption of acid green 1 dye using a co-precipitation method. Lin et al. [19] synthesized ZrO_2_/carbon aerogel (CA) composites with different amounts of monoclinic and tetragonal ZrO_2_ crystallites. The adsorption capacity of ZrO_2_/CA materials for cationic RhB dyes is 95.42 mg·g^−1^. SiO_2_ is a porous material with low density, good thermal stability, chemically stable properties, and excellent adsorption properties, and is widely used as a carrier xerogel for adsorption [20,21]. Shishmakov et al. [22] synthesized ZrO_2_-SiO_2_ xerogels through the hydrolysis of a mixture of tetrabutoxyzirconium and tetraethoxysilane in a desiccator in a vapor of a 15% aqueous NH_3_ atmosphere. Viter [23] synthesized mixed oxide dry gels of ZrO_2_-SiO_2_ from ZrOCl_2_-8H_2_O and tetraethylorthosilicate [Si(OEt)_4_] by the sol–gel method. The synthesized gels had a substantially increased porosity with specific surface areas of 140–630 m^2^·g^−1^ and pore volumes of 0.087–0.441 cm^3^·g^−1^. Huang et al. [24] prepared a SiO_2_-ZrO_2_ xerogel using TEOS as a silicon source and ZrO(NO)_3_·2H_2_O as Zr source. Its specific surface area reached up to 525.6 m^2^·g^−1^ after 600 °C heat treatment, with an average pore size of 8.5 nm and a pore volume of 1.16 cm^3^·g^−1^ and an RhB adsorption capacity of about 119 mg·g^−1^ at pH = 4 and a contact time of 4 h.

So far, studies on hydrophilic ZrO_2_-SiO_2_ xerogels are relatively common [25], however, when preparing ZrO_2_-SiO_2_ xerogels, a large amount of -OH groups are generated during the hydrolysis of TEOS, which can easily cause shrinkage or even cracking during the synthesis of composite xerogels and destroy their pore structure and affecting the adsorption performance. In this regard, the modification of hydrophilic ZrO_2_-SiO_2_ composite xerogels with methyl hydrophobicity is a worthwhile approach to explore. For this reason, in this paper, the hydrophobic modification was performed by introducing methyl groups based on the synthesis of hydrophilic ZrO_2_-SiO_2_ composite xerogels, and the characterization results of two samples and different influencing factors on the adsorption performance of RhB were compared.

For this, RhB was selected as the adsorbent and hydrophilic and hydrophobic ZrO_2_-SiO_2_ xerogels were prepared in this paper. The xerogels were characterized by FTIR, XRD, SEM, and N_2_ adsorption–desorption. The adsorption performance of the hydrophilic and hydrophobic ZrO_2_-SiO_2_ xerogels on RhB was analyzed under the influence of different dosages, pH, and adsorption times and temperatures. In addition, the adsorption kinetics and adsorption isotherms of hydrophobic ZrO_2_-SiO_2_ xerogel were investigated. Prepare for the removal of more diverse dyes in the future.

## 2. Experimental Section

### 2.1. Sol Preparation

#### 2.1.1. Preparation of ZrO_2_ Sol

The ZrO_2_ sols were prepared by sol–gel method using zirconium nitrate pentahydrate (Zr(NO_3_)_4_·5H_2_O, Tianjin Fuchen Chemical Reagent Co., Ltd., Tianjin, China) as the precursor. A certain amount of Zr(NO_3_)_4_ solution was added to the three-mouth flask, and 0.2 mol·L^−1^ of C_2_H_2_O_4_ solution was added to the Zr (NO_3_)_4_ solution drop by drop with the volume ratio of Zr(NO_3_)_4_/C_2_H_2_O_4_ = 3/2. After the temperature of the water bath reached 50 °C, 30% (*v*/*v*) of propanetriol (GL, p.a. grade, Tianjin Kemiou Chemical Reagent Co., Ltd., Tianjin, China) was added drop by drop and stirred strongly for 3 h, and the clarified and transparent ZrO_2_ sol was obtained after aging at 25 °C for 12 h.

For the ZrO_2_ sol, the reactions are as Formulas (1)–(3):

(1) $$\mathrm{Zr}{\left({\mathrm{NO}}_{3}\right)}_{4}+4H_{2}O\to \mathrm{Zr}{\left(\mathrm{OH}\right)}_{4}+4{\mathrm{HNO}}_{3}$$

(2) $$\equiv \mathrm{Zr}–\mathrm{OH}+\mathrm{OH}–\mathrm{Zr}\equiv \;\to \;\mathrm{Zr}–O–\mathrm{Zr}\equiv +H_{2}O$$

(3) $$\mathrm{Zr}{\left(\mathrm{OH}\right)}_{4}+2C_{2}H_{2}O_{4}\equiv \;\to \;=\mathrm{Zr}{\left(C_{2}O_{4}\right)}_{2}+4H_{2}O$$

#### 2.1.2. Preparation of Hydrophilic SiO_2_ Sol

The molar composition of chemical regents preparing the hydrophilic SiO_2_ sol was TEOS:EtOH:H_2_O:HNO_3_ = 1.0:8.0:7.2:0.085. Ethyl orthosilicate (TEOS, p.a. grade, Xi’an chemical reagent Co. Ltd., Xi’an, China) and anhydrous ethanol (EtOH, p.a. grade, Tianjin Branch Micro-Europe Chemical Reagent Co., Ltd., Tianjin, China) were mixed as a hydrophilic solution and placed in an ice water bath. The mixture of H_2_O and nitric acid (HNO_3_, p.a. grade, Sichuan Xilong Reagent Co., Ltd., Xilong, China) was added dropwise under a magnetic stirrer for 30 min. After the dropwise addition, the mixture was stirred at reflux for 3 h at 60 °C and cooled to obtain the hydrophilic SiO_2_ sol.

#### 2.1.3. Preparation of Hydrophobic SiO_2_ Sol

Methyltriethoxysilane (MTES, grade 98%, Hangzhou Guibao Chemical Co. Ltd., Hangzhou, China) was used as a hydrophobic agent, and according to the molar ratio of TEOS:MTES:EtOH:H_2_O:HNO_3_ = 1.0:0.8:8.0:7.2:0.085, it was first mixed completely with MTES, TEOS, and EtOH, placed in an ice-water bath, and stirred fully under a magnetic stirrer for 30 min before adding dropwise H_2_O and nitric acid mixture, and after the dropwise addition, it was stirred at 60 °C under reflux for 3 h and cooled to obtain methyl-modified hydrophobic SiO_2_ sol.

#### 2.1.4. Preparation of Hydrophilic and Hydrophobic ZrO_2_-SiO_2_ Sols

According to the Zr/Si molar ratio of 0.15, a mixture of ZrO_2_ sol was added drop by drop into the freshly prepared SiO_2_ sols and methyl-modified SiO_2_, respectively. After stirring strongly for 60 min at 25 °C, the hydrophilic ZrO_2_-SiO_2_ and hydrophobic ZrO_2_-SiO_2_ sols were obtained, respectively.

The co-hydrolysis and condensation reactions of TEOS and MTES are as Formulas (4)–(9):

Hydrolysis reactions:

(4) $$\mathrm{Si}{\left({\mathrm{OCH}}_{2}{\mathrm{CH}}_{3}\right)}_{4}+{\mathrm{nH}}_{2}O\to \mathrm{Si}{\left({\mathrm{OCH}}_{2}{\mathrm{CH}}_{3}\right)}_{4-n}{\left(\mathrm{OH}\right)}_{n}+{\mathrm{nCH}}_{3}{\mathrm{CH}}_{2}\mathrm{OH}$$

(5) $${\mathrm{CH}}_{3}–\mathrm{Si}{\left({\mathrm{OCH}}_{2}{\mathrm{CH}}_{3}\right)}_{3}+{\mathrm{nH}}_{2}O\to {\mathrm{CH}}_{3}–\mathrm{Si}{\left({\mathrm{OCH}}_{2}{\mathrm{CH}}_{3}\right)}_{3-n}{\left(\mathrm{OH}\right)}_{n}+{\mathrm{nCH}}_{3}{\mathrm{CH}}_{2}\mathrm{OH}$$

Condensation reactions:

(6) $$\equiv {\mathrm{SiOCH}}_{2}{\mathrm{CH}}_{3}+\mathrm{HO}–\mathrm{Si}\equiv \;\to \mathrm{Si}–O–\mathrm{Si}\equiv +{\;\mathrm{CH}}_{3}{\mathrm{CH}}_{2}\mathrm{OH}$$

(7) $$=\mathrm{Si}({\mathrm{CH}}_{3})–{\mathrm{OCH}}_{2}{\mathrm{CH}}_{3}+\mathrm{HO}–\mathrm{Si}\equiv \;\to \;=\mathrm{Si}({\mathrm{CH}}_{3})–O–\mathrm{Si}\equiv +{\;\mathrm{CH}}_{3}{\mathrm{CH}}_{2}\mathrm{OH}$$

(8) $$\equiv \mathrm{Si}–\mathrm{OH}+\mathrm{HO}–\mathrm{Si}\equiv \;\to \;\equiv \mathrm{Si}–O–\mathrm{Si}\equiv +H_{2}O$$

(9) $$=\mathrm{Si}({\mathrm{CH}}_{3})–\mathrm{OH}+\mathrm{HO}–\mathrm{Si}\equiv \;\to \;=\mathrm{Si}({\mathrm{CH}}_{3})–O–\mathrm{Si}\equiv +H_{2}O$$

The reactions between ZrO_2_ and hydrophilic SiO_2_, hydrophobic SiO_2_ sols are as Formulas (10) and (11):

(10) $$\equiv \mathrm{Zr}–\mathrm{OH}+\mathrm{HO}–\mathrm{Si}\equiv \;\to \;\equiv \mathrm{Zr}–O–\mathrm{Si}\equiv +H_{2}O$$

(11) $$\equiv \mathrm{Zr}–\mathrm{OH}+{\mathrm{CH}}_{3}{\mathrm{CH}}_{2}O–\mathrm{Si}\equiv \;\to \;\equiv \mathrm{Zr}–O–\mathrm{Si}\equiv +{\mathrm{CH}}_{3}{\mathrm{CH}}_{2}\mathrm{OH}$$

### 2.2. Preparation of Xerogels

The as-prepared ZrO_2_, hydrophilic and hydrophobic SiO_2_, and hydrophilic and hydrophobic ZrO_2_-SiO_2_ sols were placed in the Petri dishes, respectively, and dried at 50 °C. The formed gels were ground into powders and then roasted in a program-controlled high-temperature furnace at 400 °C for 2 h under N_2_ atmosphere with a heating rate of 1 °C·min^−1^.

### 2.3. Characterization

FTIR spectra were recorded on a PerkinElmer Spotlight 400 and Frontier spectrometer and KBr pellets were prepared and taken over a wavelength range of 400–4000 cm^−1^. XRD patterns were performed on a RigakaD/max 2200 X-ray diffractometer, using CuK*α* radiation and scanning 2θ from 4 to 90°, operated at 40 kV and 40 mA. The JSM-6700F SEM was used to investigate the morphology of the samples. X-ray photoelectron spectroscopy (XPS) was used to analyze the surface chemical composition of the hydrophilic and hydrophobic ZrO_2_-SiO_2_ xerogels. (ESCALAB250xi, Thermo Scientific, Waltham, MA, USA). The pressure in the analysis chamber was maintained at 3.0 × 10^−7^ Pa. The binding energy values were referenced to the C (1 s) line situated at 284.6 eV. N_2_ absorption–adsorption isotherms and pore size distributions were obtained from the ASAP2020 Plus automatic analyzer, micromeritics. Molecular structure models were constructed using ChemDraw software 18.0 (PerkinElmer Corporation, Waltham, MA, USA, Software license: Sijie Marking Software Co., Ltd., Suzhou, China).

### 2.4. Water Adsorption Measurement

To examine the hydrophobicity of the two ZrO_2_-SiO_2_ xerogels, the hydrophilic and hydrophobic ZrO_2_-SiO_2_ samples were aged in a constant temperature and humidity chamber of 25 °C and 75% RH. During the aging period, the samples were weighed, and the mass changes and water adsorption were calculated.

### 2.5. Adsorption Performance Test

The effects of adsorption time, adsorption temperature, dosage, and pH on the removal of RhB were investigated by adsorption experiments. A certain amount of rhodamine B (RhB, Relative molecular weight (Mw) = 479.01, λ_max_ = 554 nm, Tianjin Comio Chemical Reagent Co., Ltd., Tianjin, China) dye was dissolved in deionized water to prepare a standard solution of 140 mg·L^−1^. The Zeta potential of the xerogels was measured using a Nano-ZS tester manufactured in the UK and the average of three measurements was taken.

The hydrophilic and hydrophobic ZrO_2_-SiO_2_ xerogels (0.05 g) were separately dispersed in the above RhB solution (70 mL) under vigorous stirring (700 rpm) to investigate the effects of adsorption time, adsorption temperature and pH, and the two xerogels with the amount of 0.01, 0.05, 0.1, 0.15, and 0.2 g were separately dispersed into the RhB solution (150 mL) to investigate the effect of dosage. The adsorption experiments were conducted at 25, 35, and 45 °C, respectively. The solid–liquid mixture was separated by centrifugation. The liquid phase was analyzed using a UV-V spectrophotometer at a wavelength of 554 nm. The RhB concentrations were then calculated from the calibration curve.

The adsorption amount *q*_e_ of RhB by the hydrophilic and hydrophobic ZrO_2_-SiO_2_ xerogels ate calculated by Equation (12):

(12) $$q_{e}=\frac{\;\left(C_{0}-C_{e}\right)V}{W}$$

The removal rate of RhB in water is calculated by Equation (13):

(13) $$R=\frac{\;C_{0}-C_{e}}{C_{0}}\times 100\%$$

where *C*_0_ and *C*_e_ (mg·L^−1^) is the initial and equilibrium concentration of RhB, respectively. *V* (L) is the volume of the solution. *W* (g) is the dry mass of the xerogels.

### 2.6. Desorption Performance Test

The hydrophilic and hydrophobic ZrO_2_-SiO_2_ xerogels were rinsed with anhydrous ethanol at room temperature, and the process was repeated 6 times for multiple cycles of adsorption and desorption to test the reproducibility of the prepared xerogel adsorbents.

## 3. Results and Discussion

### 3.1. FTIR Analysis

**Figure 2:**
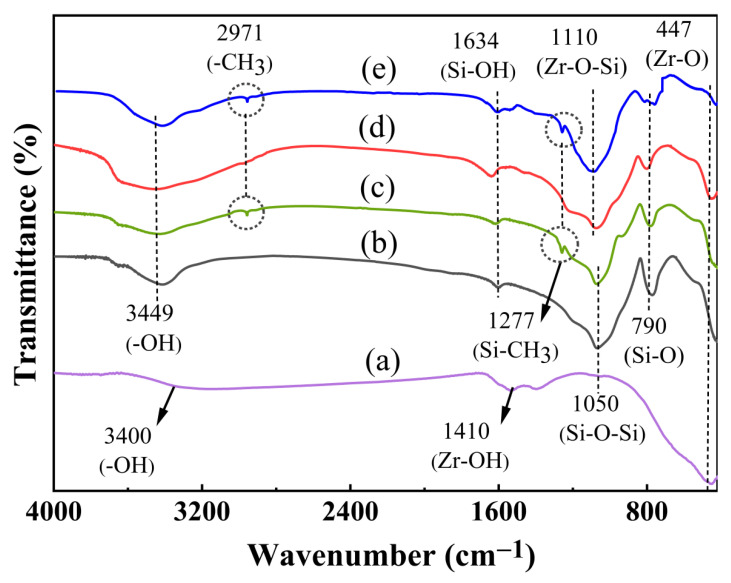
FTIR spectra comparison of (a) ZrO_2_, (b) hydrophilic SiO_2_, (c) hydrophobic SiO_2_, (d) hydrophilic ZrO_2_-SiO_2,_ and (e) hydrophobic ZrO_2_-SiO_2_ xerogels.

The functional groups of ZrO_2_, hydrophilic and hydrophobic SiO_2_, and hydrophilic and hydrophobic ZrO_2_-SiO_2_ xerogels were investigated using FTIR spectra, shown in Figure 2. In Figure 2, the absorption peak at around 3449 cm^−1^ is the stretching and bending vibration of the -OH group caused by the absorption of water, the characteristic peak at 1634 cm^−1^ is caused by the Si-OH bonds [26] of the xerogel, the peaks located around 790 cm^−1^ are related to the stretching vibration of the Si-O bond [27], and these sets of absorption peaks co-occur in the hydrophilic and hydrophobic SiO_2_, hydrophilic and hydrophobic ZrO_2_-SiO_2_ xerogels. As can be seen in Figure 2, the absorption peak of Si-O-Si in hydrophilic and hydrophobic SiO_2_ xerogels is 1050 cm^−1^ [28]. In hydrophilic and hydrophobic ZrO_2_-SiO_2_ xerogels, the absorption peaks of Zr-O-Si appeared at 1100 cm^−1^, and the absorption peak at 1110 cm^−1^ is due to the presence of Zr perturbing the three-dimensional asymmetric stretching vibrations of Si-O-Si forming a Zr-O-Si bond, indicating the chemical bonding of SiO_2_ to ZrO_2_ particles [29]. The methyl hydrophobic modification of the xerogel is achieved by depleting the -OH group in the Si-OH bond on the xerogel surface and introducing the hydrophobic group -CH_3_ to form the Si-CH_3_ bond. Compared with SiO_2_ and hydrophilic ZrO_2_-SiO_2_, the hydrophobic SiO_2_ and ZrO_2_-SiO_2_ exhibited -CH_3_ absorption peak at 2971 cm^−1^ and Si-CH_3_ absorption peak at 1277 cm^−1^, indicating the successful modification of the methyl hydrophobic groups. The characteristic peaks of -OH and Zr-OH can be seen in ZrO_2_ xerogel at 3400 cm^−1^ and 1410 cm^−1^, respectively, along with an absorption peak of Zr-O at 447 cm^−1^. In addition, it can be seen from Figure 2 that the absorption peaks at 3449 cm^−1^ and 1634 cm^−1^ of hydrophobic xerogel are slightly weaker than hydrophilic xerogel, whereas the presence of this indicates the consumption of -OH and the formation of Si-CH_3_. According to infrared spectrum analysis, combined with the reaction equations, there are Si-O-Si, Zr-O-Si, Si-OH, Zr-OH, and Zr-O groups on the surfaces of hydrophilic and hydrophobic ZrO_2_-SiO_2_ xerogels. In addition, the surface of the hydrophobic ZrO_2_-SiO_2_ sample also contains Si-CH_3_ groups.

### 3.2. Phase Structure Analysis

**Figure 3:**
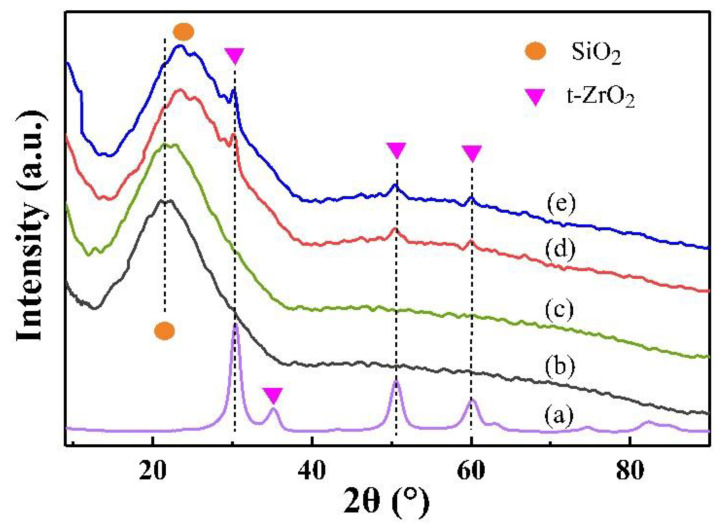
XRD pattern of (a) ZrO_2_, (b) hydrophilic SiO_2_, (c) hydrophobic SiO_2_, (d) hydrophilic ZrO_2_-SiO_2_, and (e) hydrophobic ZrO_2_-SiO_2_ xerogels.

Figure 3 shows the XRD patterns of ZrO_2_, hydrophilic and hydrophobic SiO_2_, and hydrophilic and hydrophobic ZrO_2_-SiO_2_ xerogels. The tetragonal phase ZrO_2_ diffraction peaks at 2θ = 30.57, 35.26, 50.41, and 60.63° can be seen in ZrO_2_. In the hydrophilic and hydrophobic SiO_2_, and hydrophilic and hydrophobic ZrO_2_-SiO_2_ xerogels, the distinctive diffraction peaks all appear between 2θ = 20~30°, related to the presence of amorphous SiO_2_. In contrast, the silicon diffraction peak of the hydrophilic and hydrophobic SiO_2_ xerogels appears at 2θ = 20.88°, whereas the silicon diffraction peaks of the hydrophilic and hydrophobic ZrO_2_-SiO_2_ xerogels appear at 2θ = 24.08°, mainly because the introduced zirconium atoms replaced some silicon atoms and formed the Zr-O-Si bonds, which lead to a decrease in SiO_2_ content, the crystal diffraction peaks move to a larger angle. In hydrophilic and hydrophobic ZrO_2_-SiO_2_ xerogels, because of the formation of Zr-O-Si bonds, the tetragonal t-ZrO_2_ crystallographic surfaces appear in the diffraction peaks at 2θ = 30.57°, 50.41°, and 60.63° [30]. However, the intensity of the diffraction peak is weak and the dispersion broadens, indicating that the nuclei of t-ZrO_2_ are produced with relatively low crystallinity [31]. Comparing before and after the modification, the structures of the xerogels phases were similar and the diffraction peaks did not differ significantly.

**Figure 4:**
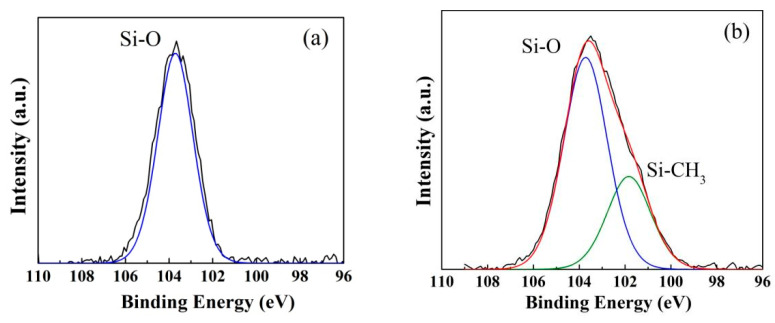
Si 2p spectra of XPS analysis for the (**a**) hydrophilic and (**b**) hydrophobic ZrO_2_-SiO_2_ xerogels.

The surface chemical composition of the hydrophilic and hydrophobic ZrO_2_-SiO_2_ xerogels was analyzed by XPS as shown in Figure 4. Figure 4a shows that the Si 2p spectra of the hydrophilic ZrO_2_-SiO_2_ sample consist of the main component with a binding energy of 103.69 eV due to the Si-O species on the sample surface. The Si 2p spectra of the hydrophobic ZrO_2_-SiO_2_ sample contain two peaks as shown in Figure 4b. It is reasonable to assign the peak lying at the binding energy of about 103.72 eV mainly to the Si-O species, and the peak lying at the binding energy of around 101.79 eV to the Si-CH_3_. It is seen that the chemical environment of the Si on the two sample surfaces changes significantly with the addition of MTES during the sol preparation. Compared with the hydrophilic sample, the morphology of the Si 2p peak of hydrophobic ZrO_2_-SiO_2_ xerogel changed significantly, and the Si 2p spectral peak became wider and moved to the direction of low bond energy, which indicated that the chemical bond structure of Si 2p changed significantly after hydrophobic modification by methyl and the bond energy of Si decreased.

### 3.3. Pore Structure Analysis

**Figure 5:**
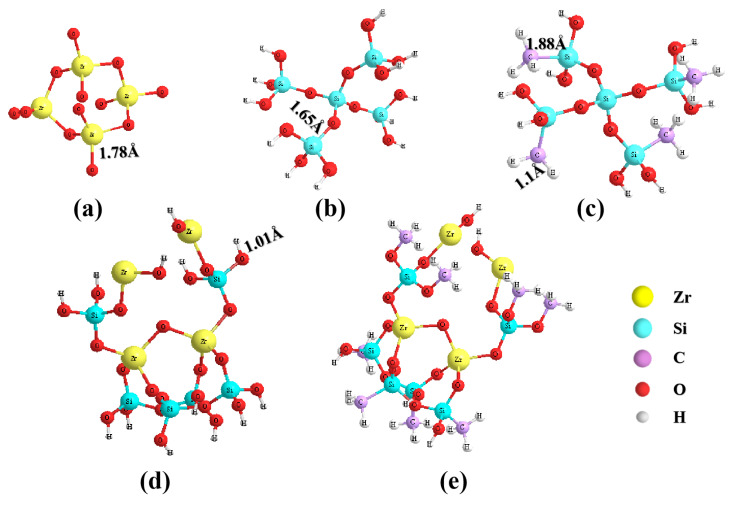
Molecular structural models of (**a**) ZrO_2_, (**b**) hydrophilic SiO_2_, (**c**) hydrophobic SiO_2_, (**d**) hydrophilic ZrO_2_-SiO_2_, and (**e**) hydrophobic ZrO_2_-SiO_2_ xerogels.

**Figure 6:**
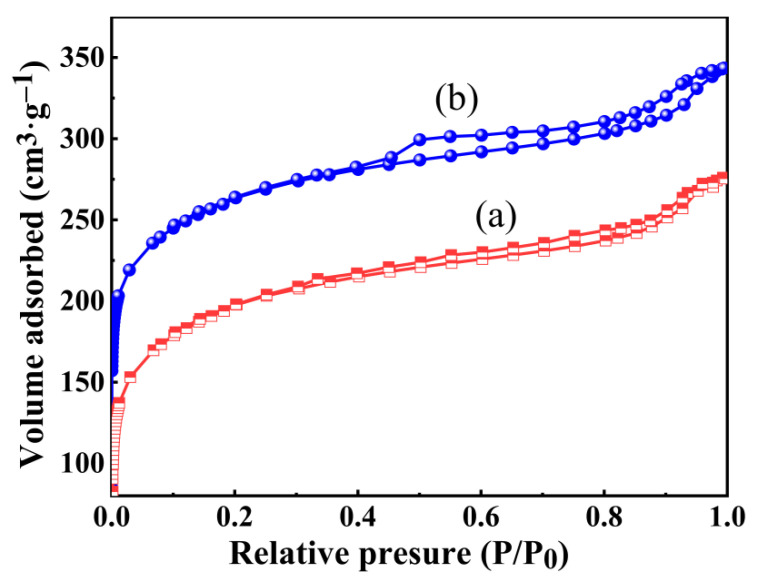
The N_2_ adsorption-desorption isotherms for the (a) hydrophilic and (b) hydrophobic ZrO_2_-SiO_2_ xerogels.

**Figure 7:**
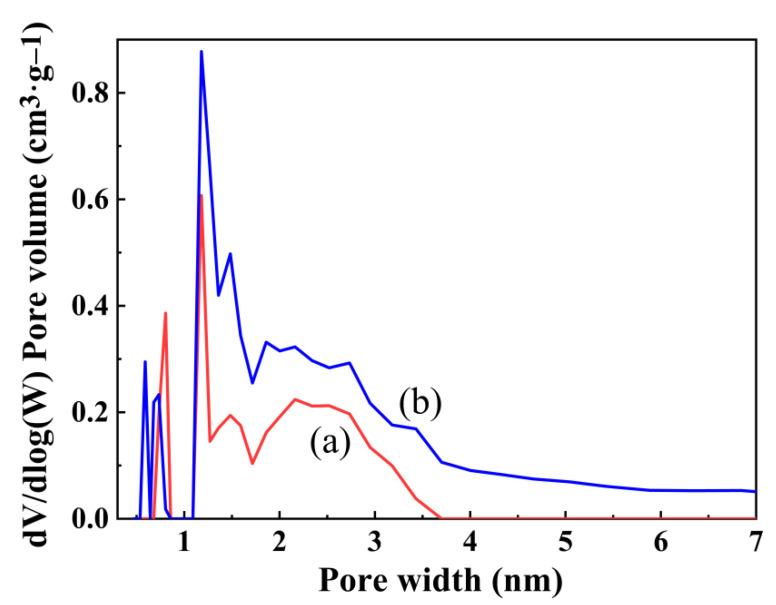
The corresponding pore size distribution curves for the (a) hydrophilic and (b) hydrophobic ZrO_2_-SiO_2_ xerogels.

Figure 5 shows the molecular structure of ZrO_2_, SiO_2_, hydrophobic SiO_2_, hydrophilic ZrO_2_-SiO_2_, and hydrophobic ZrO_2_-SiO_2_. Figure 6 shows the N_2_ adsorption–desorption curves of hydrophilic and hydrophobic ZrO_2_-SiO_2_ xerogels. From Figure 6, the isotherms of hydrophilic and hydrophobic ZrO_2_-SiO_2_ xerogels showed the type IV isotherms of Brunauer–Deming–Deming–Teller (BDDT) classification, which exhibited a microporous structure at P/P_0_ < 0.4 and a hysteresis loop near P/P_0_ = 0.4, indicating the presence of mesopores in the xerogels. It can be seen from Figure 6 that the N_2_ adsorption of hydrophobic ZrO_2_-SiO_2_ xerogel is greater than that of hydrophilic ZrO_2_-SiO_2_. Compared with hydrophilic ZrO_2_-SiO_2_, the N_2_ adsorption of hydrophobic ZrO_2_-SiO_2_ xerogel increased by 30.96%. Figure 7 shows the pore size distributions of the hydrophilic and hydrophobic ZrO_2_-SiO_2_ xerogels. In Figure 7, it can be seen that the pore size distribution of hydrophilic and hydrophobic ZrO_2_-SiO_2_ xerogels are similar, and the pore distribution ranges from the micropore to the mesoporous region. In the graph, the total pore volume, specific surface area, and pore size of the hydrophobic ZrO_2_-SiO_2_ xerogel are greater than those of the hydrophilic ZrO_2_-SiO_2_ xerogel, mainly because the bond lengths of Si-CH_3_ groups in the hydrophobic ZrO_2_-SiO_2_ xerogel are longer than those of Si-OH in hydrophilic ZrO_2_-SiO_2_ xerogel (Figure 5), the bond lengths of Si-C (1.88 Å) and C-H (1.1 Å) are longer than those of Si-O (1.65 Å) and O-H (1.01 Å), respectively.

The pore structure parameters of hydrophilic and hydrophobic ZrO_2_-SiO_2_ xerogels are shown in Table 1. The average pore size, BET surface area, and total pore volume of hydrophobic ZrO_2_-SiO_2_ xerogel are larger than those of hydrophilic ZrO_2_-SiO_2_ xerogel. Compared with the hydrophilic ZrO_2_-SiO_2_ xerogel, the specific surface area and pore capacity of the hydrophobic ZrO_2_-SiO_2_ xerogel sample increased by 62.76% and 59.26%, respectively. Generally speaking, the adsorption performance of an adsorbent depends mainly on its surface properties and the BET surface area, whereas a larger BET surface area and a suitable pore size are more favorable [27] for the adsorption of dye molecules. The hydrophobic xerogel was more advantageous.

### 3.4. SEM Analysis

**Figure 8:**
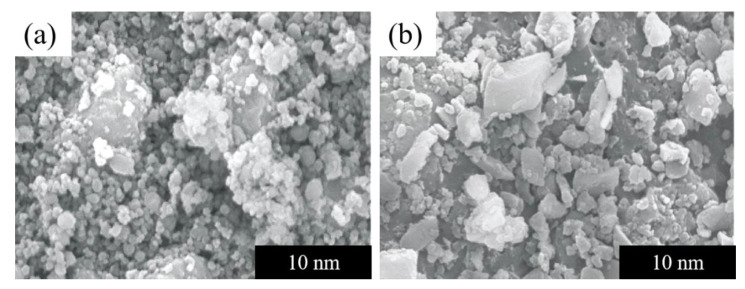
SEM comparison of (**a**) hydrophilic ZrO_2_-SiO_2_, (**b**) hydrophobic ZrO_2_-SiO_2_ xerogels.

The SEM images of hydrophilic and hydrophobic ZrO_2_-SiO_2_ xerogels are shown in Figure 8. In Figure 8, it can obviously be seen that the particle size of hydrophobic ZrO_2_-SiO_2_ xerogel is larger than that of the hydrophilic sample. As described in the pore structure analysis, this is a result of the introduction of Si-CH_3_ groups, in which the bond lengths of Si-C and C-H are longer than those of Si-O and O-H, respectively.

### 3.5. Water Absorption Analysis

**Figure 9:**
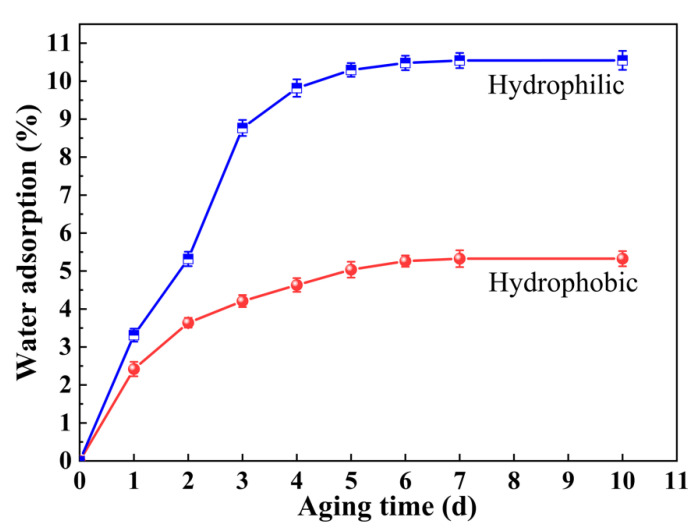
Water absorption diagram of hydrophilic ZrO_2_-SiO_2_ and hydrophobic ZrO_2_-SiO_2_ xerogels.

The water absorption of hydrophilic and hydrophobic ZrO_2_-SiO_2_ xerogels was analyzed, shown in Figure 9. As shown in Figure 9, with the increase in aging time, the water absorption of the two ZrO_2_-SiO_2_ samples gradually increased. It reached its saturated state after 6 d, and the saturated water absorption was about 10.55% and 5.33% for the hydrophilic and hydrophobic samples, respectively. Compared with the hydrophilic ZrO_2_-SiO_2_ xerogel, the water absorption of the hydrophobic sample decreased by 49.5%. The water absorption of porous materials is related to their hydrophobicity and specific surface area. In general, more hydrophilic surfaces and larger specific surface areas correspond to greater water absorption. As shown in Table 1, the specific surface area of the hydrophobic ZrO_2_-SiO_2_ xerogel is larger than that of the hydrophilic one. This means that the introduced Si-CH_3_ bonds in the hydrophobic ZrO_2_-SiO_2_ xerogel have higher hydrophobicity than the Si-OH bonds, which can lead to less water absorption.

### 3.6. Adsorption Performance Studies

#### 3.6.1. Effect of Adsorption Time and Temperature

**Figure 10:**
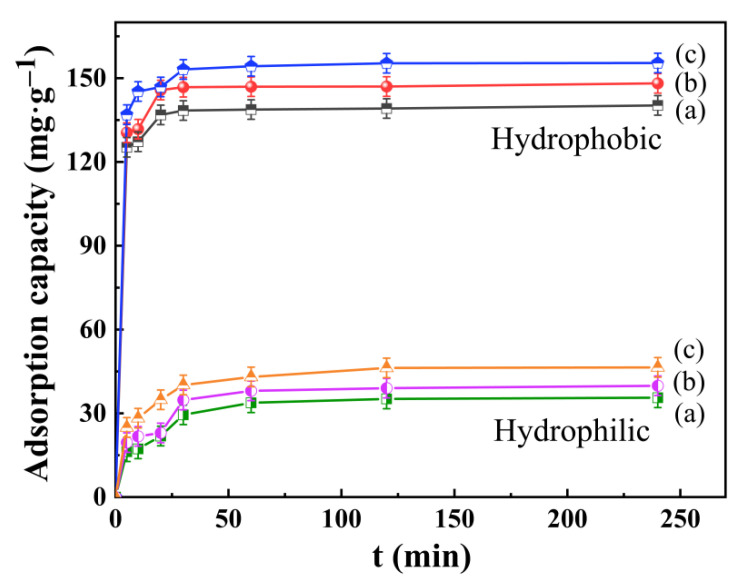
Effect of hydrophilic and hydrophobic ZrO_2_-SiO_2_ xerogels at various adsorption temperatures (a) 25 °C, (b) 35 °C, and (c) 45 °C on the adsorption capacity of RhB (pH = 7, RhB concentration = 140 mg·L^−1^).

Figure 10 shows the effect of the adsorption capacity of hydrophilic and hydrophobic ZrO_2_-SiO_2_ xerogel with the change in adsorption time and adsorption temperature. As shown in Figure 10, the hydrophobic xerogel exhibited rapid adsorption during the first 30 min, after which it was basically in adsorption equilibrium. The hydrophilic xerogel was basically in adsorption equilibrium at 120 min. The adsorption amounts of hydrophilic and hydrophobic xerogels increased by 10.88 mg·g^−1^ and 15.18 mg·g^−1^ with increasing temperature, respectively, and reached the maximum adsorption amount at 45 °C. In addition, the adsorption capacity of the hydrophobic xerogel was increased by 70.3% relative to that of hydrophilic ZrO_2_-SiO_2_ xerogel. The results showed that the hydrophobic xerogel had a superior adsorption effect on the RhB solution. As seen in Figure 10, the adsorption process of hydrophobic ZrO_2_-SiO_2_ on RhB was endothermic, and in general, the normal adsorption process is exothermic. However, if the adsorption process is influenced by intraparticle diffusion, the adsorption capacity will increase with temperature due to the heat absorption of the diffusion process [27]. This means that increasing the temperature increases the diffusion rate of adsorbent RhB molecules between the outer boundary layer and the inner pores of the hydrophobic ZrO_2_-SiO_2_ xerogel, thus increasing the adsorption capacity of hydrophobic ZrO_2_-SiO_2_.

As the hydrophobic ZrO_2_-SiO_2_ xerogel contains methyl groups on its surface, the RhB surface is also rich in methyl groups (Figure 1), making the hydrophobic xerogel similarly soluble with RhB and providing a better adsorption effect. Therefore, the hydrophobic ZrO_2_-SiO_2_ xerogel was chosen for the subsequent experiments.

#### 3.6.2. Effect of Dosage

**Figure 11:**
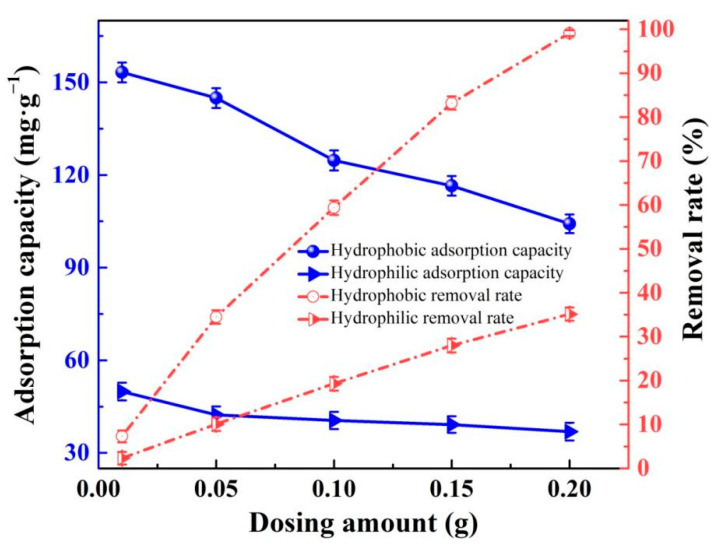
The adsorption capacities and removal rates of the hydrophilic and hydrophobic ZrO_2_-SiO_2_ xerogels to RhB at various dosages (pH = 7, contact time = 120 min, RhB concentration = 140 mg·L^−1^; T = 25 °C).

Figure 11 shows the adsorption capacity for the unit mass of RhB by hydrophilic and hydrophobic ZrO_2_-SiO_2_ xerogels with various dosages. It can be seen from Figure 11 that the removal rate of both samples increased with the increase in the dosage, and the removal rate of the hydrophobic xerogel was 24.43% higher than that of the hydrophilic one at the dosage of 0.05 g. In addition, the adsorption capacity for the unit mass of both xerogels reached the maximum value at the dosage of 0.01 g and decreased gradually with the increase in dosage. When the dose was increased from 0.01 g to 0.05 g, the adsorption per unit mass decreased by 7.6 mg·g^−1^ and 8.3 mg·g^−1^ for hydrophilic and hydrophobic xerogels, respectively. The total amount of adsorption increased with increasing dose, whereas the adsorption per unit mass decreased gradually. The reason is that as the amount of RhB remains unchanged, with the gradual increase in the amount of adsorbent, the number of adsorption active sites in the system increases and the adsorption rate accelerates, which leads to a decrease in the utilization of adsorption active sites per unit mass of adsorbent, resulting in a lower adsorption amount for unit mass. The adsorption capacity for the unit mass of hydrophobic ZrO_2_-SiO_2_ xerogel was higher than those of hydrophilic ZrO_2_-SiO_2_ xerogel because the introduced -CH_3_ group increased the specific surface area and the contact area with RhB solution, and the adsorption capacity of the hydrophobic xerogel increased by 207.0% and 242.5% relative to the hydrophilic one at the dosage of 0.01 g and 0.05 g, respectively.

#### 3.6.3. Effect of pH

**Figure 12:**
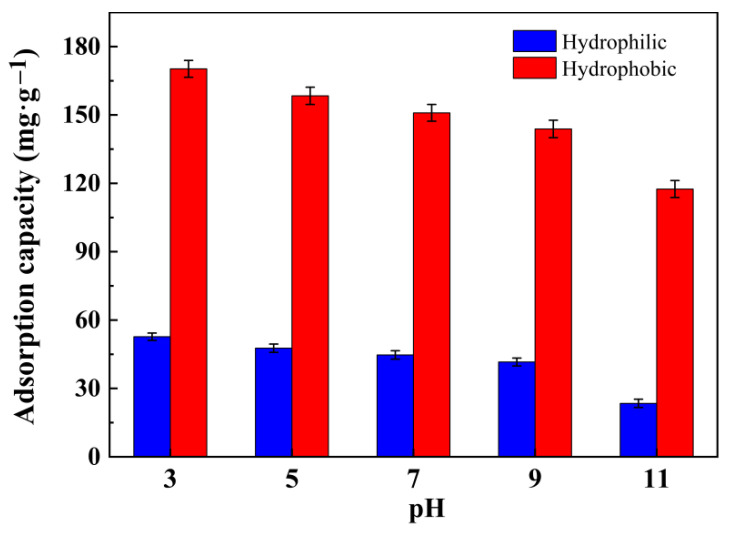
Adsorption capacities of RhB for the hydrophilic and hydrophobic ZrO_2_-SiO_2_ xerogels at different pH (dosage = 0.05 g, contact time = 120 min, RhB concentration = 140 mg·L^−1^, T = 25 °C).

**Figure 13:**
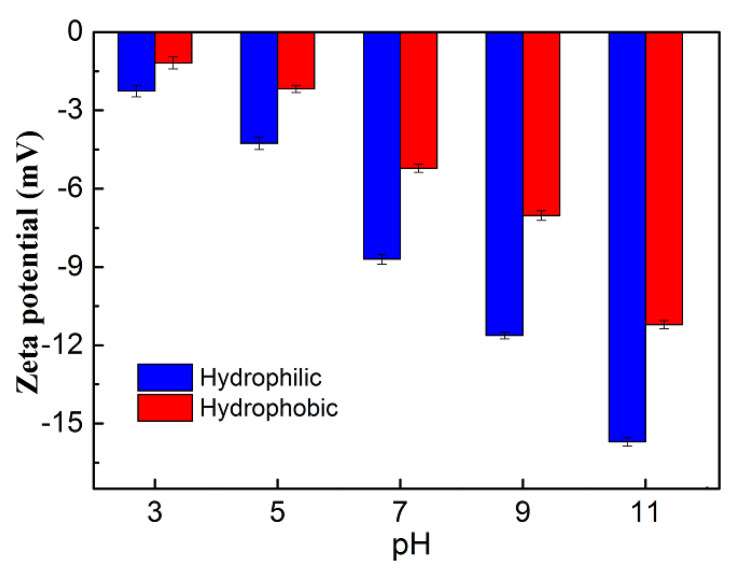
Effect of solution pH on the zeta potentials of hydrophilic and hydrophobic ZrO_2_-SiO_2_ xerogels.

In the adsorption experiments, the solution pH is an important factor in controlling the adsorption process and may have a great influence on the final adsorption effect, because it affects the surface charge of the adsorbent in the solution as well as the degree of ionization and molecular structure of the dyes [27]. The adsorption capacities of RhB for the hydrophilic and hydrophobic ZrO_2_-SiO_2_ xerogels at different pH were shown in Figure 12. In Figure 12, the variation of adsorption capacity for the two samples showed similar trends. With the increases in pH value, the adsorption capacities of RhB for the hydrophilic and hydrophobic ZrO_2_-SiO_2_ xerogels tended to decrease gradually, which had a maximum of 52.42 and 169.23 mg·g^−1^ at pH = 3, respectively. When the pH value increased from 3 to 11, the adsorption capacities of RhB for the hydrophilic and hydrophobic samples decreased by 55.5% and 31.0%, respectively. The effect of solution pH on the zeta potentials of the two adsorbents is shown in Figure 13. At pH = 3–11, the two adsorbents both had a negative zeta potential and their absolute values increased with the increasing pH value. Furthermore, the absolute value of the zeta potential of the hydrophilic sample was greater than that of the hydrophobic one at the same pH value. RhB is a cationic basic dye and its acid dissociation constant pKa is at about 3.2. When pH = 3, the RhB is presented in cationic and monomeric molecular form, there is electrostatic attraction existing between RhB and the two adsorbents. Thus, the organic skeleton of RhB easily can enter into the pores of the xerogels. When pH = 5–11, the RhB exists in the form of zwitterions. At the time, they are not easy to enter into the pore structure. In the process of adsorption of RhB by the adsorbents, the electrostatic mechanism is not only the mechanism of dye adsorption in this system but also the interaction between the adsorbents and dye molecules via hydrogen bonds and hydrophobic structures. The hydrophobic ZrO_2_-SiO_2_ xerogel has the hydrophobic Si-CH_3_ groups, whereas the hydrophilic sample does not. This further increases the adsorption capacity of hydrophobic xerogel to RhB. Furthermore, the results indicate that the acidic environment is favorable for the adsorption of RhB by the hydrophilic and hydrophobic ZrO_2_-SiO_2_ xerogels.

### 3.7. Adsorption Kinetic Analysis

**Figure 14:**
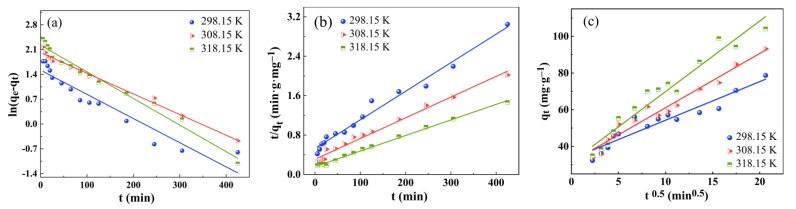
Adsorption kinetics of RhB adsorbed by hydrophobic ZrO_2_-SiO_2_ xerogel: (**a**) pseudo–first-order kinetic plots, (**b**) pseudo–second-order kinetic plots, and (**c**) intra-particle diffusion plots (dosage = 0.05 g, pH = 3, RhB concentration = 140 mg·L^−1^, contact time = 120 min, T = 298.15, 308.15, and 318.15 K).

The adsorption kinetic analysis process is designed to investigate the kinetics and mechanism of adsorption of RhB by hydrophobic ZrO_2_-SiO_2_ xerogel, and it includes pseudo–first-order dynamics, pseudo–second-order dynamics, and intra-particle diffusion models. The experimental data were fitted by a kinetic sorption model [32,33], which was calculated and fitted using Equations (14) and (15). The fitting results are shown in Table 2 and Figure 14a–c.

The calculation of the pseudo–first-order dynamics is given in equation:

(14) $$\ln\left(q_{e}-q_{t}\right)=\ln q_{e}-K_{1}t$$

The calculation of the pseudo–second-order dynamics is given in equation:

(15) $$\frac{t}{q_{t}}=\frac{1}{K_{2}q_{e}^{2}}+\frac{t}{q_{e}}$$

The adsorption rate is usually controlled by membrane diffusion, intra-particle diffusion, or both of them. The first–second-order and pseudo–second-order kinetic models cannot determine the role of intra-particle diffusion in the adsorption dynamics process. In order to identify whether the intra-particle diffusion is a rate-limiting step in the adsorption kinetics process or not, the experimental data is further fitted by the intra-particle diffusion model [34], and the calculation formula is shown in (16):

(16) $$q_{t}=K_{\mathrm{di}}t^{0.5}+C$$

where *t* (min) is the adsorption time. *q*_e_ and *q*_t_ (mg·g^−1^) are the adsorption capacities at equilibrium and at time *t*, respectively. *K*_1_ (min^−1^) and *K*_2_ (g·mg^−1^·min ^−1^) are the pseudo–first-order and pseudo–second-order adsorption rate constants (min^−1^), respectively. *K*_di_ (mg·g^−1^·min^−0.5^) is the intra-particle diffusion rate constant. *C* represents the greater effect of the boundary layer on molecule diffusion.

The high values of *R*^2^ in Table 2 indicate that the adsorption of RhB onto hydrophobic ZrO_2_-SiO_2_ xerogel can be approximated more appropriately by a pseudo–second-order kinetic model. According to the pseudo–second-order model, the boundary layer resistance is not a rate-limiting step [35]. Moreover, the investigation of diffusion mechanism with the intra-particle diffusion model is important. The linear fitting result is shown in Figure 14c. The linearity is attributed to the mesopores diffusion, which is an accessible site of adsorption. The fitted lines did not pass through the origin; hence, the adsorption rate is affected by both intra-particle diffusion and film diffusion. In short, the adsorption of RhB onto hydrophobic ZrO_2_-SiO_2_ xerogel is a complex process. The related plots in Figure 14a–c indicate that the RhB adsorption reaction proceeds through three steps: the first stage is the rapid adsorption phase of the outer surface (film diffusion), the second stage is the slow adsorption stage (pore or intra-particle diffusion), and the third stage is the adsorption equilibrium stage which means the adsorption reaction is no longer carried out.

### 3.8. Adsorption Isotherm Analysis

**Figure 15:**
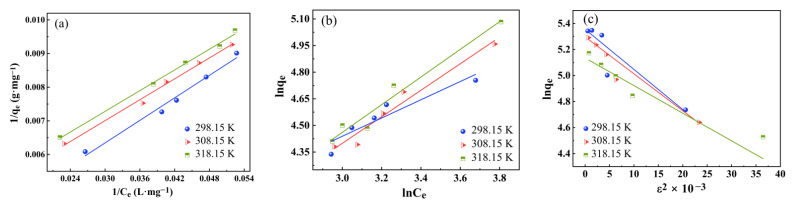
(**a**) Langmuir, (**b**) Freundlich and, (**c**) D–R adsorption isotherm of RhB onto hydrophobic ZrO_2_-SiO_2_ xerogel (dosage = 0.05 g, pH = 3, RhB concentration = 140 mg·L^−1^, contact time = 120 min, T = 298.15, 308.15 and 318.15 K).

In order to understand the interactions of RhB with the hydrophobic ZrO_2_-SiO_2_ xerogel, the Langmuir [36], Freundlich [37] and Dubinin–Radushkevih (D–R) models [38] are used at 298.15, 308.15 and 318.15 K, respectively. To describe the adsorption behavior of RhB onto the hydrophobic ZrO_2_-SiO_2_ xerogel as follow:

The calculation of the Langmuir model is given in Equation (17):

(17) $$\frac{1}{q_{e}}=\frac{1}{q_{m}bC_{e}}+\frac{1}{q_{m}}$$

where *b* (L·mg^−1^) is the Langmuir constant. *q*_m_ is the maximum monolayer adsorption capacity (mg·g^−1^), the Langmuir isotherm plots of 1/*q*_e_ versus 1/*C*_e_ are given in Figure 15a, and the parameters are exhibited in Table 3.

Dimensionless constant (*R*_L_) [39] was used to identify the feasibility and favorability of the adsorption process. *R*_L_ is calculated in each case using the Equation (18):

(18) $$R_{L}=\frac{1}{{1+bC}_{0}}$$

The values of *R*_L_ reveal that the isotherms are irreversible (*R*_L_ = 0), unfavorable (*R*_L_ > 1), favorable (0 < *R*_L_ < 1), or linear (*R*_L_ = 1) [40].

The calculation of the Freundlich isotherm model is given in Equation (19):

(19) $${\ln q}_{e}{=\ln K}_{F}+\frac{1}{n}{\ln C}_{e}$$

where *K*_F_ (L·g^−1^) and *n* are the Freundlich constant related to adsorption capacity and adsorption intensity, respectively. The Freundlich isotherm plots of ln*q*_e_ versus ln*C*_e_ are shown in Figure 15b and the parameters are exhibited in Table 3.

In order to determine the type of adsorption reaction, the calculation of the Dubinin–Radushkevich (D–R) isotherm model (Figure 15c) is given in Equations (20)–(22):

(20) $${\ln q}_{e}=\ln q_{D}-B\cdot \varepsilon^{2}$$

(21) $$\varepsilon =RT\ln\left(1+\frac{1}{C_{e}}\right)$$

(22) $$E=\frac{1}{\sqrt{2B}}$$

where *q*_D_ is the theoretical saturation capacity, mg·g^−1^. *B* is the adsorption energy constant, mol^2^·J^−2^. *ε* is the Polanyi potential energy, J·mol^−1^. *R* is the ideal gas constant, 8.314 J·mol^−1^·K^−1^. *T* is the thermodynamic temperature, K. *E* is the average adsorption energy, kJ·mol^−1^.

From Figure 15 and Table 3, it is seen that the Langmuir isotherm model is more suitable for the RhB adsorption onto hydrophobic ZrO_2_-SiO_2_ xerogel with *R*^2^ values greater than 0.99 for both and that the adsorption process may be monolayer adsorption. Maximum adsorption capacity increased with an increase in the temperature from 139.77 mg·g^−1^ to 173.53 mg·g^−1^, revealing the endothermic nature of the adsorption process. As can be seen in Table 3, for the dimensionless constants, *R*_L_ values (0.1123~0.1231) are all less than 1, which indicates that RhB adsorption of hydrophobic ZrO_2_-SiO_2_ xerogel is favorable. In addition, the adsorption constant 0 < (1/*n*) < 1 of the Freundlich model indicates that the hydrophobic ZrO_2_-SiO_2_ xerogel has inhomogeneous surface properties and RhB is easily adsorbed on the surface of the hydrophobic ZrO_2_-SiO_2_ xerogel. With the increase in adsorption temperature, the adsorption energy (*E*) increases. It is indicated that the adsorption of RhB onto hydrophobic ZrO_2_-SiO_2_ xerogel is an endothermic behavior. As can be seen from Table 3, the calculated *E* values are found to be less than 8 kJ·mol^−1^, indicating that the adsorption process is mainly physical adsorption [41]. The results of this experiment also confirm the better prospect of hydrophobic ZrO_2_-SiO_2_ composites for the removal of dye wastewater.

Table 4 shows the comparison of Langmuir parameters for different adsorbents from different researchers. From Table 4, it can be seen that the R^2^ of these adsorbent materials is close to 1, which indicates that the adsorption is relatively good. The *R*_L_ values are also all less than 1, which indicates that the adsorption is irreversible. The data show that the maximum adsorption capacity of the hydrophilic adsorbent in the fourth cited paper is 177.7 mg·g^−1^, however, its equilibrium adsorption capacity is only about 120 mg·g^−1^ and the preparation method is different, and the equilibrium adsorption capacity in this paper is as high as 169.23 mg·g^−1^. This means that the maximum adsorption capacity of the hydrophobic SiO_2_-ZrO_2_ xerogel prepared in this paper is higher than the average value of most adsorbents. Therefore, it indicates that the hydrophobically modified adsorbent in this paper is successful.

## 4. Reusability of Xerogel Adsorbent

**Figure 16:**
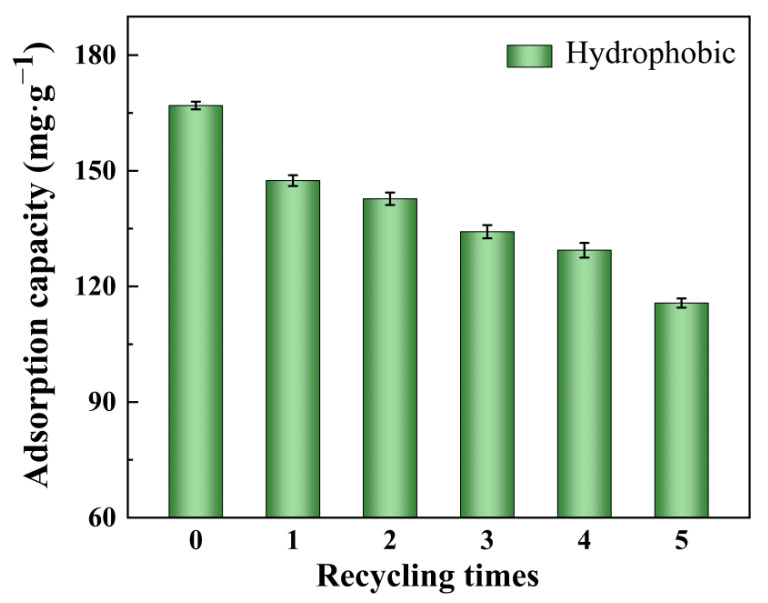
Recycling performance of hydrophobic ZrO_2_-SiO_2_ xerogel at different recycling times.

In addition to the high adsorption capacity, the regeneration and recycling properties of the xerogel are important for a potential application. Figure 16 shows the recycling performance of hydrophobic ZrO_2_-SiO_2_ xerogel at different recycling times. As shown in Figure 16, the xerogel samples were easily separated from the RhB solution with ethanol. After adsorption, the xerogel was washed with ethanol at room temperature and reused to adsorb RhB again. This regeneration procedure was repeated five times, and the adsorption capacity of the hydrophobic ZrO_2_-SiO_2_ xerogel decreased from 169.23 mg·g^−1^ to 115.74 mg·g^−1^, and the adsorption capacity decreased by 31.6%. The results show that the hydrophobic ZrO_2_-SiO_2_ xerogel has good reusability for the adsorption of RhB and can be used repeatedly many times.

## 5. Conclusions

In this paper, the hydrophilic and hydrophobic ZrO_2_-SiO_2_ xerogels were prepared by the sol–gel method. The results showed that the hydrophobic Si-CH_3_ groups were introduced by MTES modification. There are Si-O-Si, Zr-O-Si, Si-OH, Si-CH_3_, Zr-OH, and Zr-O groups on the surfaces of hydrophobic ZrO_2_-SiO_2_ xerogel. The hydrophobic ZrO_2_-SiO_2_ xerogel has a larger specific surface area, mean pore size, and pore volume. A high adsorption capacity of 169.23 mg·g^−1^ for the hydrophobic ZrO_2_-SiO_2_ xerogel was achieved at 25 °C and pH = 3. The adsorption data fit well with the pseudo–second-order dynamics model and the isothermal adsorption curve fitting showed that the adsorption process of hydrophobic ZrO_2_-SiO_2_ on RhB was both monolayer adsorption and non-uniform adsorption, and the isothermal adsorption model at different temperatures was consistent with the Langmuir isotherm model. The results from the D–R model indicate the adsorption of RhB by hydrophobic ZrO_2_-SiO_2_ xerogel is mainly physical adsorption, accompanied by a spontaneous endothermic process. In future work, we will further investigate the adsorption performance of the hydrophobic ZrO_2_-SiO_2_ xerogel to other dyes and mixed dyes, comparing the similarities and differences in the adsorption of single-component and multi-component dye molecules.

## Figures and Tables

**Table 1:** Pore structure parameters of the hydrophilic and hydrophobic ZrO_2_-SiO_2_ xerogels.

Samples	BET Surface Area (m^2^·g^−1^)	Average Pore Size (nm)	V_total_ (STP) (cm^3^·g^−1^)
hydrophilic ZrO_2_-SiO_2_	310.13	2.16	0.27
hydrophobic ZrO_2_-SiO_2_	504.78	2.35	0.43

**Table 2:** Adsorption kinetic parameters of RhB on hydrophobic ZrO_2_-SiO_2_ xerogel at various temperatures.

Kinetic Model	Kinetic Parameters	Temperature (K)		
		298.15	308.15	318.15
*q*_e_		169.23	170.05	178.98
Pseudo–first-order	*K*_1_	0.0068	0.0058	0.0075
	*R* ^2^	0.9112	0.9886	0.9768
Pseudo–second-order	*K*_2_	0.0083	0.0063	0.0071
	*R* ^2^	0.9805	0.9832	0.9961
intra-particle diffusion	*K*_di_	2.1038	2.9532	3.8449
	*R* ^2^	0.9015	0.9594	0.9483

**Table 3:** Adsorption isotherm parameters of RhB on hydrophobic ZrO_2_-SiO_2_ xerogel at various temperatures.

Adsorption Isotherm	Isothermal Parameters	Temperature (K)		
		298.15	308.15	318.15
Langmuir	*b*	0.0031	0.0039	0.0042
	*q*_m_	139.77	154.64	173.53
	*R* ^2^	0.9924	0.9939	0.9961
	*R*_L_	0.1231	0.1191	0.1123
Freundlich	*K*_F_	18.1367	8.6576	8.3704
	1/*n*	0.5138	0.7471	0.7789
	*R* ^2^	0.8826	0.9647	0.9631
D–R	*E*	4.0285	4.1893	4.7584
	*R* ^2^	0.9302	0.9951	0.8438

**Table 4:** Langmuir parameters of materials for RhB adsorption from various researchers.

Material Type	*R* ^2^	*q*_m_	*R*_L_
Montmorillonite [42]	0.9864	42.19	0.309
Kaolinite [14]	0.98	46.08	0.94
Carbon xerogel [43]	0.9976	147.1	0.058
Hydrophilic ZrO_2_-SiO_2_ xerogel [24]	0.998	177.7	0.843
Hydrophobic ZrO_2_-SiO_2_ xerogel (this work)	0.9961	173.53	0.1123

## Data Availability

Not applicable.
