# Correction: Liu, Y.; Yang, J. Hydrophobic Modification of ZrO_2_-SiO_2_ Xerogel and Its Adsorption Properties to Rhodamine B. *Gels* 2022, *8*, 675

**DOI:** 10.3390/gels9010031

**Published:** 2022-12-30

**Authors:** Yan Liu, Jing Yang

**Affiliations:** School of Urban Planning and Municipal Engineering, Xi’an Polytechnic University, Xi’an 710048, China

In the original publication [1], there were some mistakes in the Sections 2.1.1–2.1.4, 2.5, 3.2 and 3.6.2. The authors apologize for any inconvenience caused.

(1) Changes in Section 2.1.1. Preparation of ZrO_2_ Sol

In paragraph 1 lines 3–4, the following changes are made.

We remove “and oxalic acid (C_2_H_2_O_4_·2H_2_O, p.a. grade, Tianjin Hedong Hongyan Chemical Reagent Co., Ltd., Tianjin, China) as the solvent”, because oxalic acid was not used as the solvent.

(2) Changes in Section 2.1.2. Preparation of Hydrophilic SiO_2_ Sol

In paragraph 1 line 2, the following changes are made.

“TEOS:MTES:EtOH:H_2_O:HNO_3_ = 1.0:0.8:8.0:0.72:0.085” should be corrected to “TEOS:EtOH:H_2_O:HNO_3_ = 1.0:8.0:7.2:0.085”, because it comprises hydrophilic SiO_2_ sol; MTES is not in it, and the H_2_O/TEOS molar ratio is 7.2 and not 0.72.

(3) Changes in Section 2.1.3. Preparation of Hydrophobic SiO_2_ Sol

In paragraph 1 line 3, the following changes are made.

“TEOS:MTES:EtOH:H_2_O:HNO_3_ = 1.0:0.8:8.0:0.72:0.085” should be corrected to “TEOS:MTES:EtOH:H_2_O:HNO_3_ = 1.0:0.8:8.0:7.2:0.085”, because the H_2_O/TEOS molar ratio is 7.2 and not 0.72.

(4) Changes in Section 2.1.4. Preparation of Hydrophilic and Hydrophobic ZrO_2_-SiO_2_ Sols.

In paragraph 2 lines 7–13, the following changes are made.

The original reaction Equations (7)–(11) should be corrected, because some symbols for double or triple bonds were incorrect, and they are stated as follows.

(7) $$\equiv \mathrm{Si}\left({\mathrm{CH}}_{3}\right)\text{-}{\mathrm{OCH}}_{2}{\mathrm{CH}}_{3}+\mathrm{HO}\text{-}\mathrm{Si}\equiv \;\to \;\mathrm{Si}\left({\mathrm{CH}}_{3}\right)\text{-}O\text{-}\mathrm{Si}\equiv +{\;\mathrm{CH}}_{3}{\mathrm{CH}}_{2}\mathrm{OH}$$

(8) $$\equiv \mathrm{Si}\text{-}\mathrm{OH}+\mathrm{HO}\text{-}\mathrm{Si}\equiv \;\to \;\mathrm{Si}\text{-}O\text{-}\mathrm{Si}\equiv +{\;H}_{2}O$$

(9) $$=\mathrm{Si}\left({\mathrm{CH}}_{3}\right)\text{-}\mathrm{OH}+\mathrm{HO}\text{-}\mathrm{Si}\equiv \;\to \;\mathrm{Si}\left({\mathrm{CH}}_{3}\right)\text{-}O\text{-}\mathrm{Si}\equiv +{\;H}_{2}O$$

(10) $$\mathrm{Zr}\text{-}\mathrm{OH}+\mathrm{HO}\text{-}\mathrm{Si}\;\to \;\mathrm{Zr}\text{-}O\text{-}\mathrm{Si}+H_{2}O$$

(11) $$\mathrm{Zr}\text{-}\mathrm{OH}+{\mathrm{CH}}_{3}{\mathrm{CH}}_{2}O\text{-}\mathrm{Si}\to \mathrm{Zr}\text{-}O\text{-}\mathrm{Si}+{\mathrm{CH}}_{3}{\mathrm{CH}}_{2}\mathrm{OH}$$

The corrected reactions should be as follows.

(7) $$=\mathrm{Si}\left({\mathrm{CH}}_{3}\right)–{\mathrm{OCH}}_{2}{\mathrm{CH}}_{3}+\mathrm{HO}–\mathrm{Si}\equiv \;\to =\mathrm{Si}\left({\mathrm{CH}}_{3}\right)–O–\mathrm{Si}\equiv +{\;\mathrm{CH}}_{3}{\mathrm{CH}}_{2}\mathrm{OH}$$

(8) $$\equiv \mathrm{Si}–\mathrm{OH}+\mathrm{HO}–\mathrm{Si}\equiv \;\to \;\equiv \mathrm{Si}–O–\mathrm{Si}\equiv +{\;H}_{2}O$$

(9) $$=\mathrm{Si}\left({\mathrm{CH}}_{3}\right)–\mathrm{OH}+\mathrm{HO}–\mathrm{Si}\equiv \;\to =\mathrm{Si}\left({\mathrm{CH}}_{3}\right)–O–\mathrm{Si}\equiv +{\;H}_{2}O$$

(10) $$\equiv \mathrm{Zr}–\mathrm{OH}+\mathrm{HO}–\mathrm{Si}\equiv \;\to \;\equiv \mathrm{Zr}–O–\mathrm{Si}\equiv +H_{2}O$$

(11) $$\equiv \mathrm{Zr}–\mathrm{OH}+{\mathrm{CH}}_{3}{\mathrm{CH}}_{2}O–\mathrm{Si}\equiv \;\to \;\equiv \mathrm{Zr}–O–\mathrm{Si}\equiv +{\;\mathrm{CH}}_{3}{\mathrm{CH}}_{2}\mathrm{OH}$$

(5) Changes in Section 2.5. Adsorption Performance Test

In paragraph 1 line 3, the following changes are made.

“λ_max_ = 552 nm” should be corrected to “λ_max_ = 554 nm”. It is a clerical error because the λ_max_ used is 554 nm; moreover, the mentioned measure wavelength is 554 nm in the next paragraph. 

(6) Changes in Section 2.5. Adsorption Performance Test

In paragraph 2 lines 1–2, the following changes are made.

The volumes of the RhB solution are different in the adsorption performance tests, so the sentence “The hydrophilic and hydrophobic ZrO_2_-SiO_2_ xerogels (0.01, 0.05, 0.1, 0.15, 0.2 g) were separately dispersed in the above RhB solution (50 mL) under vigorous stirring (700 rpm).” should be corrected to the following:

“The hydrophilic and hydrophobic ZrO_2_-SiO_2_ xerogels (0.05 g) were separately dispersed in the above RhB solution (70 mL) under vigorous stirring (700 rpm) to investigate the effects of adsorption time, adsorption temperature and pH, and the two xerogels with the amount of 0.01, 0.05, 0.1, 0.15, and 0.2 g were separately dispersed into the RhB solution (150 mL) to investigate the effect of dosage.” 

(7) Changes in Section 3.2. Phase Structure Analysis

In the original publication [1], Figure 4a was the Si 2p spectra of the XPS analysis for the hydrophobic SiO_2_ xerogels and not for the hydrophilic ZrO_2_-SiO_2_ xerogel, and they are shown as follows.

The corrected Figure 4 should be the following.

**Figure 4:**
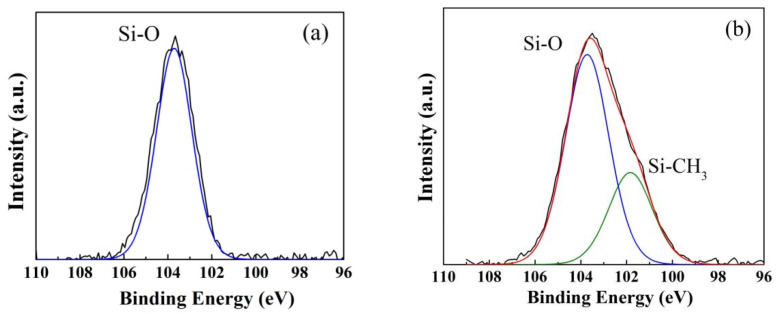
Si 2p spectra of XPS analysis for the (**a**) hydrophilic and (**b**) hydrophobic ZrO_2_-SiO_2_ xerogels.

(8) Changes in Section 3.6.2. Effect of Dosage

In paragraph 1 lines 3–5, the following changes are made.

In the dosage test experiment, the removal rates in the original publication [1] were not calculated correctly; they were recalculated. Thus, the sentence “whereas the maximum removal rate of the hydrophobic xerogel was 23.03% higher than that of the hydrophilic one.” should be corrected to the following:

“and the removal rate of the hydrophobic xerogel was 24.43% higher than that of the hydrophilic one at the dosage of 0.05 g.” 

(9) Changes in Section 3.6.2. Effect of Dosage

In paragraph 1 lines 17–20, the following changes are made.

The values 66.1% and 71.5% are the proportions of hydrophilic xerogel lower than the hydrophobic one and not the percentages of hydrophobic xerogel greater than the hydrophilic one; their calculation formulas are different. Thus, the sentence “and the adsorption capacity of the hydrophobic xerogel increased by 66.1% and 71.5% relative to the hydrophilic at 0.01 g and 0.05 g, respectively. Therefore, the highest utilization rate was achieved at the dosage of 0.05 g.” should be corrected to the following:

“and the adsorption capacity of the hydrophobic xerogel increased by 207.0% and 242.5% relative to the hydrophilic one at the dosage of 0.01 g and 0.05 g, respectively.”

(10) Changes in Section 3.6.2. Effect of Dosage

In the dosage test experiment, the removal rates in the original publication [1] were not calculated correctly; they were recalculated. Thus, Figure 11 should be corrected.

The original Figure 11 is shown as follows.

**Figure 11:**
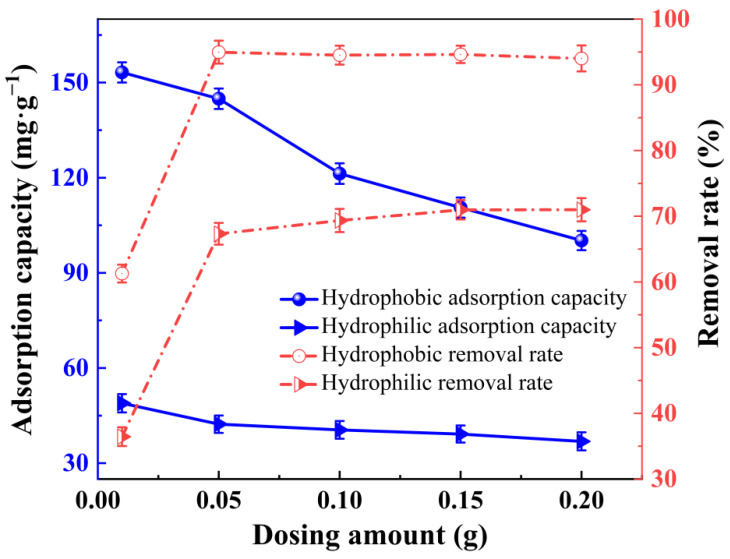
Effect of hydrophilic and hydrophobic ZrO_2_-SiO_2_ xerogels on the adsorption capacity and removal rate for unit mass of RhB at various dosages (pH = 7, contact time = 120 min, RhB concentration = 140 mg·L^−1^; T = 25 °C).

The corrected Figure 11 is now the following.

**Figure 11:**
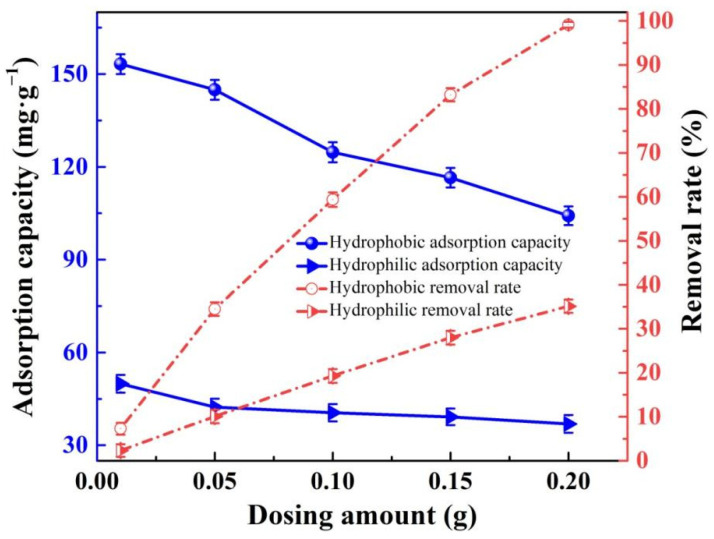
The adsorption capacities and removal rates of the hydrophilic and hydrophobic ZrO_2_-SiO_2_ xerogels to RhB at various dosages (pH = 7, contact time = 120 min, RhB concentration = 140 mg·L^−1^, T = 25 °C).

The authors state that the scientific conclusions are unaffected. This correction was approved by the Academic Editor. The original publication has also been updated.

## Figures and Tables

**Figure 4:**
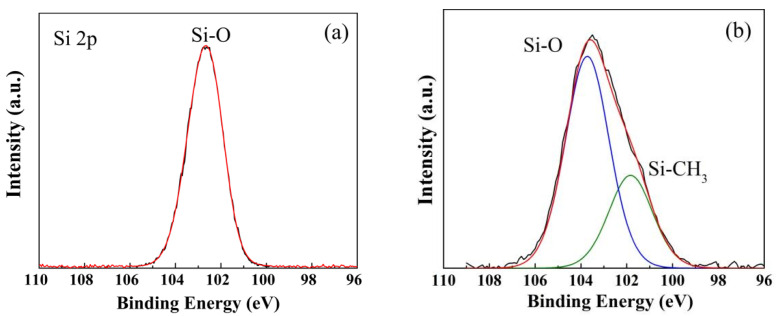
Si 2p spectra of XPS analysis for the (**a**) hydrophilic and (**b**) hydrophobic ZrO_2_-SiO_2_ xerogels.
